# Dietary behaviour change intervention for managing sarcopenic obesity among community-dwelling older people: a pilot randomised controlled trial

**DOI:** 10.1186/s12877-023-04327-w

**Published:** 2023-09-26

**Authors:** Yue-Heng Yin, Justina Yat Wa Liu, Maritta Välimäki

**Affiliations:** 1 School of Nursing, Nanjing Medical University, Nanjing, China; 2 School of Nursing, The Hong Kong Polytechnic University, Hong Kong SAR, China; 3 Faculty of Medicine, Department of Nursing Science, University of Turku, Turku, Finland

**Keywords:** Sarcopenic obesity, Dietary behavior change, Randomised Controlled Trial, Protein, Caloric restriction

## Abstract

**Background:**

The effects of dietary intervention in managing sarcopenic obesity are controversial, and behavior change techniques are lacking in previous studies which are important for the success of dietary intervention. This study aimed to evaluate the feasibility and preliminary effects of a dietary behaviour change (DBC) intervention on managing sarcopenic obesity among community-dwelling older people in the community.

**Methods:**

A two-armed, RCT was conducted. Sixty community-dwelling older adults (≥ 60 years old) with sarcopenic obesity were randomised into either the experimental group (n = 30), receiving a 15-week dietary intervention combined with behaviour change techniques guided by the Health Action Process Approach model, or the control group (n = 30), receiving regular health talks. Individual semi-structured interviews were conducted with 21 experimental group participants to determine the barriers and facilitators of dietary behaviour changes after the intervention.

**Results:**

The feasibility of the DBC intervention was confirmed by an acceptable recruitment rate (57.14%) and a good retention rate (83.33%). Compared with the control group, the experimental group significantly reduced their body weight (*p* = 0.027, *d* = 1.22) and improved their dietary quality (*p* < 0.001, *d* = 1.31). A positive improvement in handgrip strength (from 15.37 ± 1.08 kg to 18.21 ± 1.68 kg), waist circumference (from 99.28 ± 1.32 cm to 98.42 ± 1.39 cm), and gait speed (from 0.91 ± 0.02 m/s to 0.99 ± 0.03 m/s) was observed only in the experimental group. However, the skeletal muscle mass index in the experimental group decreased. The interview indicated that behaviour change techniques enhanced the partcipants’ compliance with their dietary regimen, while cultural contextual factors (e.g., family dining style) led to some barriers.

**Conclusion:**

The DBC intervention could reduce body weight, and has positive trends in managing handgrip strength, gait speed, and waist circumference. Interestingly, the subtle difference between the two groups in the change of muscle mass index warrants futures investigation. This study demonstrated the potential for employing dietary behaviour change interventions in community healthcare.

**Trial registration:**

Registered retrospectively on ClinicalTrailas.gov (31/12/2020, NCT04690985).

**Supplementary Information:**

The online version contains supplementary material available at 10.1186/s12877-023-04327-w.

## Background

Low muscle mass/function and excess adiposity, known as sarcopenic obesity, often coexist in older adults [1–3]. Sarcopenic obesity is triggered by a variety of unhealthy lifestyles (e.g., a sedentary life and an unhealthy diet) and physiological factors (e.g., a decline in growth hormones, insulin resistance, an increase in oxidative stress), that occur during the ageing process [4–6]. Sarcopenic obesity can significantly increase the risk of developing cardio-metabolic diseases, fatigue, physical disability, institutionalization, and mortality [7–9] when compared with either obesity or sarcopenia alone. The prevalence of sarcopenic obesity in China can be up to 20.4% in women and 27.0% in men [10].

A systematic review [11] of 12 randomised controlled studies (n = 863) on people with sarcopenic obesity showed that the diagnostic criteria of sarcopenic obesity in previous studies were various with great heterogeneity, which leads to lacking representativeness of participants and might affect the true effects of the interventions. Additionally, effective intervention forms on managing sarcopenic obesity are still not clear, whereas the role of nutritional interventions cannot be ignored. Adequate protein intake is essential for building muscles [4, 12, 13], and caloric restriction is effective at reducing fat mass [11].

Among the different nutritional interventions, dietary modification is a good way of managing sarcopenic obesity and may produce longer-term benefits than oral supplements alone [5], which is also recommended by the Dietary Guidelines for Chinese [14] and Americans [15]. To date, only two studies have been conducted by applying pure nutritional intervention and produce inconsistent results [16, 17], the effective doses of protein intake and caloric restriction for sarcopenic obese older people are still controversial, we have to synthesise evidence from relevant studies.

Two RCTs showed that a dose of 1.2 g/kg of body weight/day of protein intake could achieve significant increases in muscle mass for sarcopenic older adults [16, 17]. Additional guidelines and recommendations [18–20] indicated that 0.8–1.5 g/kg body weight/day of protein is recommended for older people who want to maintain optimal muscle function as they age. Therefore, a dose of 1.2–1.5 g/kg body weight/day of protein intake could be useful for sarcopenic obese older people during caloric restriction. With regard to caloric restrictions for older people, it is noteworthy that caloric restrictions varied significantly between studies. Stringent caloric restrictions are harmful and could exacerbate muscle loss in older people with sarcopenic obesity [5]. A two-year RCT with 218 participants showed that a moderate 11.9% reduction in calorie intake could promote a sustained average weight reduction, simultaneously safeguarding muscle mass [21]. Therefore, a diet of a 12% reduction in calorie intake and protein intake of 1.2–1.5 g/kg body weight/day may lead to fat loss while preserving muscle mass.

In addition, poor adherence and high dropout rates were often reported in previous dietary interventional trials of older adults, leading to inconsistent results. Successful dietary modifications require participants to adhere to a diet regimen [22, 23]. Therefore, behaviour change techniques grounded on a tested effective theoretical model can be incorporated within a diet modification intervention to improve the adherence of participants.

The primary aims of this pilot trial is to evaluate the feasibility and acceptability of the dietary behaviour change intervention (12% caloric reduction/day + 1.2–1.5 g/kg body weight/day of protein intake) for 15 weeks among community-dwelling older adults with sarcopenic obesity. The secondary aim is to evaluate the preliminary effects of the intervention on body composition, muscle strength, and physical performance.

## Methods

This study was reported according to the Consolidated Standards of Reporting Trials (CONSORT) for randomised pilot and feasibility trials [24] (CONSORT Checklist please see Supplemental Material 1).

### Trial design

This study was conducted as a prospective, two-armed, assessor-blinded, parallel-group, pilot randomised controlled trial (RCT) with an allocation ratio of 1:1. A qualitative interview of the experimental group was conducted after the pilot RCT. This trial was conducted between Jun 2020 and Feb 2021, and it has been retrospectively registered with ClinicalTrial.gov (31/12/2020, NCT04690985).

### Participants

Participants were recruited from June 2020 to November 2020 by convenience sampling. The study was promoted by displaying the posters in three largest community health centres which provided a free annual physical health examination to all citizens from the age of 60 in Nanjing, China, and community staffs also helped introduce the research project to older people who came to receive the free physical health check. Initially, participants were identified to be overweight or obese [25] by the community staffs. Then, the screening of sarcopenia was conducted by the research assistant by referring to the consensus of Asian Sarcopenia Working Group (ASWG) [26]. The final enrolment screening was conducted by the first author (YHY) according to the inclusion and exclusion criteria. The community physician ascertained whether the participant was in suitable physical health for the study. Eligible participants were interviewed to obtain their informed consent and socio-demographic data, as well as to establish a baseline assessment prior to randomisation. Participants were screened according to the inclusion and exclusion criteria shown in the Table 1.

**Table 1:** Inclusion and exclusion criteria of the participants

Inclusion criteria	Exclusion criteria
(a) community-dwelling older people aged 60 years old or above; (b) who met the diagnostic criteria of sarcopenic obesity according to the Asian Sarcopenia Working Group [29] and China’s definition of obesity for the Chinese population [28], respectively. i) *sarcopenia refers to the fulfilment of one of the following criteria*: low handgrip strength of < 28 kg for men and < 18 kg for women, or low physical performance in the 5-time chair stand test of ≥ 12 s; ii) *obesity refers to the fulfilment of one of the following criteria*: BMI ≥ 28 kg/m^2^, or waist circumference of ≥ 85 cm in men and ≥ 80 cm in women; (c) who were able to communicate, read, and write without significant hearing and vision problems.	(a) those with any form of illness or condition that may influence food intake and digestion (such as severe heart disease, metabolic disorders, autoimmune disease, cancer); (b) having cognitive impairment (e.g., dementia), which may impede delivery of the intervention; (c) already adhering to special dietary restrictions, including a diabetes-specific diet, a vegetarian diet, or a ketogenic diet; (d) taking medications that may influence eating behaviour, digestion, or metabolism (such as weight loss medication); (e) being addicted to alcohol, which affects efforts to change dietary behaviour; (f) having any metal device implanted in their body, such as a pacemaker because low-level currents will flow through the body when doing the bioelectric impedance analysis, which may cause the device to malfunction.

### Sample size

The primary objective of a pilot study is to explore the feasibility of the study. Therefore, a formal calculation of sample size is not required [27]. Hertzog suggested that a minimum of 30 participants per group would be required for a meaningful pilot study [28]. Therefore, a total of 60 participants were recruited in this pilot study.

### Intervention

***The experimental group*** received a 15-week dietary behaviour change (DBC) programme. They were recommended to follow a moderate hypocaloric diet with adequate daily protein intake. A moderate hypocaloric diet (i.e., a 12% reduction in calories from the estimated daily energy expenditure) [21] was suggested to promote a sustained reduction in average weight while simultaneously safeguarding muscle mass. The daily energy expenditure was calculated based on the basic metabolic rate (BMR) and physical activity level. The BMR was assessed via the bioelectrical impedance analysis (BIA), and physical activity level was assessed via the International Physical Activity Questionnaire Short-form (IPAQ-SF). In addition, to compensate for the blunted anabolic responses to muscle protein synthesis, a dose of 1.2–1.5 g/kg body weight/day of protein intake was recommended [18–20].

The experimental group were taught behaviour change techniques (BCTs) developed according to the health action process approach (HAPA) model [29]. The HAPA model is a psychological, behavioural change model that is used to describe and predict improvements in health-related behaviours. The model builds a bridge between motivation and action by planning and helps the participants to successfully transform their motivation into action [29]. The model contains two phases: motivation and volition [29]. The motivation phase refers to the goal initiation phase. ‘Self-efficacy’, ‘outcome expectancies’, and ‘increased risk awareness’ are the three attributes that motivate individuals to form an intention/goal to change their unhealthy lifestyle for a healthy lifestyle. The volition phase refers to the process of implementing intentions into actual behaviour through careful planning and action execution. The HAPA model has been found to be effective in previous studies, such as in promoting physical activity [30] or healthy eating habits [31].

To help the participants implement the dietary intervention, each participant was given a guidebook (Supplemental Material 2), which was developed through an evidence-based literature review and expert consultations. The delivery of the intervention contained three phases, with six face-to-face sessions alternating with weekly telephone calls, details of the interventions is shown in the Table 2: Health Action Process Approach-based DBC intervention.

**Table 2:** Health Action Process Approach-based DBC intervention protocol

Phase	Aims	When	How	Material	Content
Intention phase (2 sessions)	To establish the participants’ awareness of sarcopenic obesity, to introduce knowledge about diets, and to motivate them into forming an intention to change their behaviour.	Week 1	One-hour face-to-face talk in Weeks 1, 2	The Dietary Guidebook	A guidebook was distributed to the participants. They were educated about the concept of sarcopenic obesity, including about the following issues: ‘What is sarcopenic obesity?’, ‘What are the health consequences of sarcopenic obesity?’, ‘What are the risk factors of sarcopenic obesity?’, ‘What are the preventive treatments for sarcopenic obesity?’, ‘What is the relationship between sarcopenic obesity and diet?’.
		Week 2			The participants were introduced to information about a balanced diet for older adults (high-quality protein and low intake of calories).
Planning phase (1 session)	To help participants transform their intention into a detailed plan and to teach them to record their daily food intake.	Week 3	One-hour face-to-face talk in Week 3	a) The Dietary Guidebook b) Electronic weighing scale c) Food model	- Helping the participants to develop a plan to eliminate their worries and establish the confidence to take actions. Dietary plans focused on two aspects: enhancing protein intake (1.2–1.5 g/kg DBW/day) and restricting calorie intake (12% reduction). The participants were also encouraged to keep a balanced diet in terms of rich food variety. - Training the participants to record their daily food intake by teaching basic food weighing and recording methods, focusing on food exchange lists. The participants were trained to acquire a basic awareness of how to identify food portions corresponding to 90 kcal (1 portion food = 90 kcal). - Pictures of single portions of different types of food were shown in the guidebook. Foods rich in high-quality protein were highlighted in the guidebook (i.e., fish, dairy products, soy products, meat, eggs). The protein dose of foods (e.g., every 100 g milk contains 3 g protein) is labeled on the picture in the guidebook, which helped the participants to establish a basic awareness of the protein amount gradually.
Action phase (3 sessions)	To help the participants to continuously execute the dietary plan.	Weeks 4–15	a) One-hour face-to-face talk in Weeks 4, 8, 12; b) Weekly telephone call	a) The Dietary Guidebook b) Food diary c) Electronic weighing scale	Four strategies were used to improve the participants’ self-efficacy in all three sessions: (1) Acquiring encouragement through the achievement of the goal and engaging in the dietary change; (2) Recovering from setbacks through sharing failures and troubleshooting; (3) Gaining successful experiences from the researcher by sharing other participant’s experiences; (4) Further clarifying the goal via continuous guidance of the plan of execution. Details of the strategies were as follows: - To self-evaluate the achievement of the goals and compliance with the action plans; - To acknowledge the accomplishments to promote a positive perceived response; - To share difficulties or obstacles during the execution of the plans; - To give suggestions and help based on the specific problems; - To share positive examples and feelings from other participants via the researcher; - To continue to refine the goals and action plans and review the outcome expectations; - To guide the participants in developing their plans for the sustainability of the action.

The intervention was delivered in the community healthcare centres by a registered nurse, who is also a qualified weight management coach from the Chinese Nutrition Society. The interventionist used an intervention checklist to ensure the fidelity of the delivery. The interventionist also checked the participants’ compliance with the intake of calories and protein according to their food diary. Each participant was given a food diary notebook, they needed to write down the food type and amount they consumed every day. Training of food recording was provided during the face-to-face meetings, and continuous guidance was provided throughout the intervention. The food diary was checked during each face-to-face meeting, and the interventionist gave the participants further suggestions based on the food diary. If the participant did not keep a food diary, a three-day food recall method, a commonly used method in nutritional studies [32], was used to assess their food intake.

***The control group*** received regular health talks to control for the effects of social interaction The control group were asked to continue with their usual dietary habits. A research assistant (RA), who was not involved in other procedures in this study, contacted the participants to offer health talks according to a standard manual. The content of the health talks was unrelated to sarcopenic obesity or diet. The number and duration of contacts for the control group were similar to those for the experimental group.

### Outcomes

Outcome measurements were conducted by a trained research assistant, who was blinded to the group allocations, at baseline (T0) and immediately after the intervention (T1).

*Feasibility of the intervention*. The feasibility outcomes were measured as rates of: recruitment (i.e., length of recruitment, recruitment rate, to determine ease of recruitment), attendance (attendance in the face-to-face sessions), retention (complete follow-up), and adherence (adherence to keeping a food diary and to dietary instructions). The adherence to keeping a food diary was rated as ‘good’, ‘moderate’, or ‘bad’, according to the following average reports on frequency: ‘6–7 days/week’, ‘3–5 days/week’, and ‘0–2 days/week’, respectively. The adherence to the dietary instructions (protein and calorie intake) was assessed based on the food diary. Compliance with adequate protein intake was measured by the percentage of people whose adequate protein intake score in the Dietary Quality Index-International (DQI-I) was 5. In addition, the average amount of daily protein intake for the participants were calculated based on the food diary. Compliance with calorie control was assessed by calculating the average number of calories consumed monthly based on the food diary and three-day food recall exercise. In addition, adverse events were recorded via the CONSORT Extension for Harms checklist [33].

*Acceptability of the intervention.* Acceptability of the intervention was assessed via individual semi-structured interviews, which were arranged after the intervention for the participants from the experimental group based on their level of adherence (low, moderate, high), with the aim to better understand the participants’ perceptions about the intervention process and to characterise the facilitators and barriers to changing their dietary behaviour. There was a total of 21 participants that received interview by according to the data saturation principle, as no more new information occurred during the interviews. The development of interview script was based on the MRC framework which contains the key points of developing and implementing an intervention. The interviews were conducted by the interventionist in a private room in the community centre.

*Preliminary effects of the intervention*. Outcomes included the parameters to reflect the condition of sarcopenic obesity: body composition that measured via bioelectrical impedance analysis (BIA) with multiple frequencies (InBody 270, Korea), which included body mass index, percentage of body fat, body fat mass, skeletal muscle mass index, skeletal muscle mass adjusted by weight, waist circumference, and body weight; handgrip strength that measured via a handheld Jamar Hydraulic Hand Dynamometer; and physical performance measured via the Short Physical Performance Battery (SPPB). Additionally, nutrition self-efficacy, dietary quality, nutritional status, and health status were also measured. The detailed descriptions of outcome measurements are listed in Table 3. Additionally, participants’ physical activity status was measured at T0 and T1, using the IPAQ-SF [34], to take into account the effects of potential confounding factors.

**Table 3:** Summary of outcome measurements

Outcomes		Measurement methods
**Feasibility**	Recruitment	Length of recruitment, recruitment rate
	Attendance	Attendance in the face-to-face sessions
	Retention	Complete follow-up
	Adherence	Adherence to keeping a food diary and to dietary instructions
**Acceptability**		Individual semi-structured interviews
**Preliminary effects**		
Body composition	Body weight (kg), body fat mass (kg), BMI (kg/m^2^), percentage of body fat, and SMI (kg/m^2^), waist circumference (cm)	Bioelectrical impedance analysis (InBody 270, Korea) Tape
Muscle strength	Handgrip strength (kg)	Handheld Jamar Hydraulic Hand Dynamometer
Physical performance	6-m gait speed (m/s)	
	SPPB	To assess a person’s physical function from three aspects [52], scores range from 0 (worst performance) to 12 (best performance). ① standing for 10 s with feet in three different positions; ② 3-m or 4-m walking speed test; ③ time to rise from a chair for five consecutive times.
Nutrition self-efficacy	HAPA Nutrition Self-efficacy Scale:	Scores range from 1 to 4 with a higher score meaning higher self-efficacy [31].
Dietary quality	DQI-I, Food diary	The DQI-I reflects the participant’s usual food consumption and nutrient intake from four aspects: variety, adequacy, moderation, and overall balance. The scores range from 0 (lowest quality) to 100 (highest quality). The calculation of daily calorie intake and protein intake were analysed by a nutrition analysis software called Food Processor ® [53, 54].
Nutritional status	MNA	The MNA score ranges from 0 to 14, with a higher score representing better nutritional status [55, 56].
Health status	SF-36	It contains eight scaled scores: vitality, physical functioning, bodily pain, general health perceptions, physical role functioning, emotional role functioning, social role functioning, and mental health. A lower score suggests greater disability, while a higher score indicates less disability [57].

Notes: BMI = body mass index; SMI = skeletal muscle mass index; SPPB = Short Physical Performance Battery; HAPA = Health Action Process Approach; DQI-I = Dietary Quality Index-International; MNA = Mini Nutritional Assessment; SF-36 = 36-item short form health survey

### Randomisation and blinding

The block randomisation method (block size = 4) was utilized, to ensure that an equally balanced number of participants were allocated to each study group (i.e., the experimental or control groups). The randomisation table was obtained from the Research Randomiser software (https://www.randomiser.org/). A random sequence code was generated by a research assistant who was not involved in the implementation of the intervention or in assessing the outcome. Allocation concealment was ensured by using sealed envelopes, and upheld until the group assignment was completed. Because of our proposed intervention structure, it was impossible to implement the steps to blind the participants. Therefore, only the outcome assessor was blinded to the group allocation throughout the whole process.

### Statistical methods

Descriptive statistics (absolute number and percentage of participants) were used to present the length of recruitment, recruitment rate, retention rate, adherence rate, and completion rate of all the measurements. The recruitment rate of 50% and the adherence rate of 60% indicated an acceptable level, and the proportion of missing data for each variable was suggested to be less than 5% [35]. The SPSS version 26.0 was used to analyse the acquired data.

For the acceptability outcomes, the semi-structured interviews were digitally audio-recorded and transcribed verbatim. NVivo 12 software was used to manage the data and help identify common codes from the transcripts. Content analysis was employed inductively to synthesise the categories and themes. Two researchers (the first author and RA) worked independently on the coding and on identifying codes by following the guideline of content analysis [36]. Both coders had received research training and had experience in coding. The bracketing strategy [37] was followed during the data analysis. The coding scheme and identified themes were discussed among the research team to achieve a consensus on the final themes.

For the analysis of the preliminary effects data, the intention-to-treat (ITT) principle was followed in the data analyses. Descriptive statistics were used to present the demographic data and the feasibility outcomes. Normality assumptions were checked for variables. The homogeneity of the two groups was examined by comparing the demographic and baseline outcomes using an independent t-test or the Mann-Whiteney U test for continuous data at baseline, and the Chi-square test or Fisher’s exact test for dichotomous data. The missing variables were caused by dropouts, which were checked by using missing completely at random (MCAR) method. The generalized estimating equation (GEE) was employed to estimate the time and group effects on the clinical outcomes measured pre- and post-intervention. Two heterogeneous variables, i.e., level of education and body height, were adjusted during the statistical analysis. Considering the covariate effects, all of the GEE analyses were adjusted for three covariates (the variables related to height, level of education, and physical activity level) by considering the significant heterogeneity between the groups. A p-value of < 0.05 was considered statistically significant.

## Results

### Characteristics of the participants

**Fig. 1:**
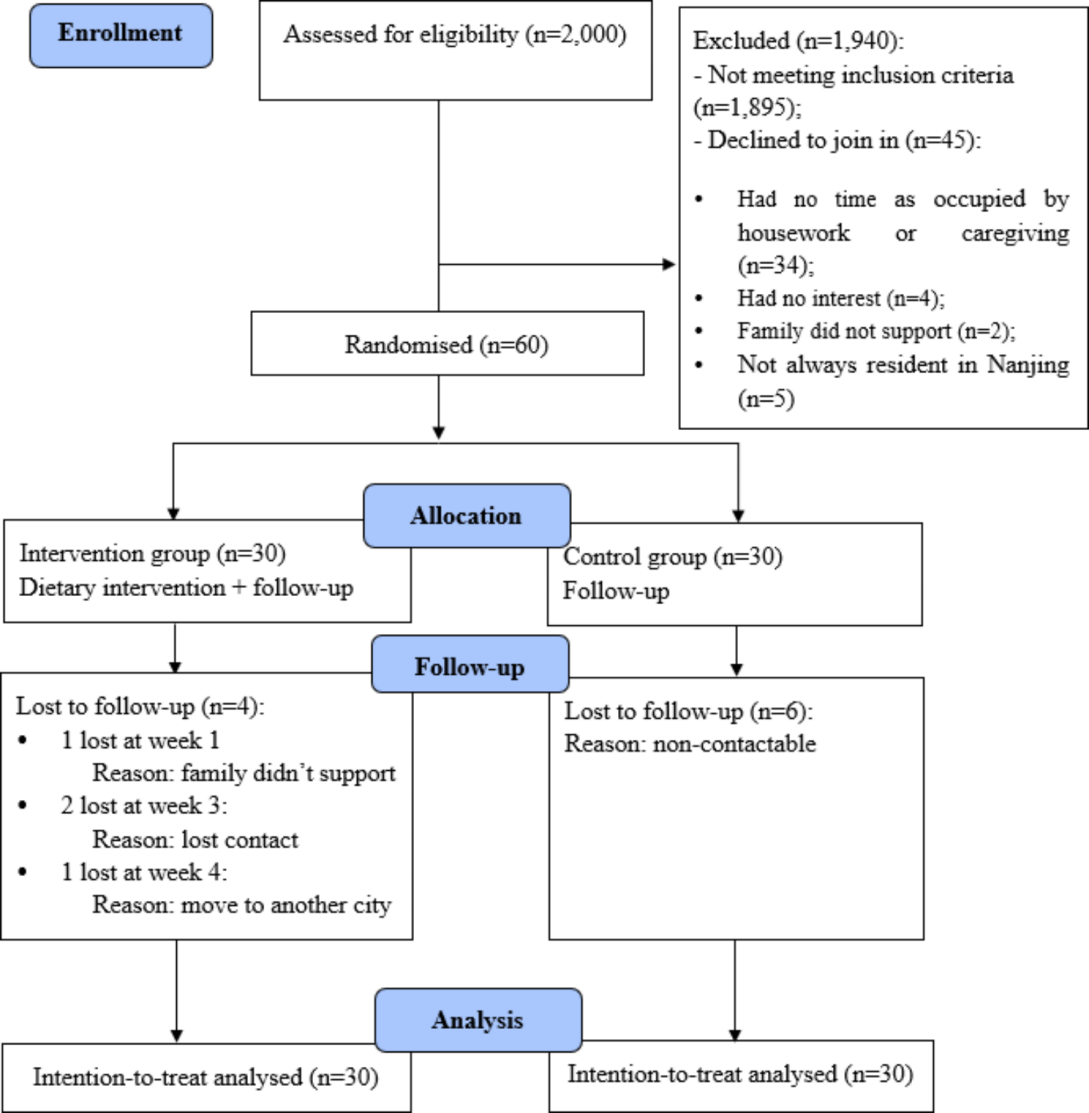
CONSORT flow chart

One hundred and five people were found to be eligible after 2,000 people were screened. Sixty of them (mean age = 68.13 ± 6.12 years old) agreed to participate (please see the CONSORT flow chart in Fig. 1). The demographic characteristics of the participants are presented in Table 4. There were no significant differences in demographics among the participants except for level of education (*χ*^*2*^ = 8.20, *p* = 0.041) and body height (*t*=-2.10, *p* = 0.035). These two heterogeneous variables were adjusted during the statistical analysis.

**Table 4:** Characteristics of the participants at baseline

Variable	Total (n = 60) *n* (%)	DBC group (n = 30) *n* (%)	Control group (n = 30) *n* (%)	χ^2^*/z*	*p*
**Age (mean, SD)**	68.13 (6.12)	68.87 (6.51)	67.40 (5.72)	− 0.70 (z)	0.481
**Gender**				2.86^a^	0.091
Female	42 (70)	18 (60)	24 (80)		
Male	18 (30)	12 (40)	6 (20)		
**Alcohol**				4.55^b^	0.327
Never	48 (80)	22 (73.33)	26 (86.67)		
Once/month	3 (5)	3 (10)	0 (0)		
2–4 times/month	3 (5)	2 (6.67)	1 (3.33)		
2–3 times/week	1 (1.67)	0 (0)	1 (3.33)		
≥ 4 times/week	5 (8.33)	3 (10)	2 (6.67)		
**Vegetarian**				.00^b^	1.000
Yes	4 (6.67)	2 (6.67)	2 (6.67)		
No	56 (93.33)	28 (93.33)	28 (93.33)		
**IPAQ**				.49^b^	0.909
Low	4 (6.67)	2 (6.67)	2 (6.67)		
Moderate	40 (66.67)	19 (63.33)	21 (70)		
High	16 (26.67)	9 (30)	7 (23.33)		
**Morbidity**				10.47^a^	0.063
Hypertension	30 (50)	12 (40)	18 (60)		
Hyperlipidemia	14 (23.3)	9 (30)	5 (16.7)		
Fatty liver	23 (38.3)	14 (46.7)	9 (30)		
Others	13 (21.7)	10 (33.3)	3 (10)		
None	8 (13.3)	4 (13.3)	4 (13.3)		

Notes: DBC = dietary behaviour change; SD = standard deviation;

IPAQ = International physical activity questionnaire

^a^ Chi-square test

^b^ Fisher’s exact test

### Feasibility of the intervention

The recruitment process lasted for around six months. The recruitment rate was 57.1% (60/105), and the overall retention rate was 83.3% (50/60) (see Fig. 1). The response rate of all the questionnaires among the participants who fully involved the trial was 100%. The percentage of people who attended the face-to-face sessions at least five out of six times was 73.3%, and the percentage of those whose rate of adherence to keeping a food diary was moderate or above was 26.7%. The rate of compliance with adequate protein intake was 66.7%, and the participants had a protein intake of 1.3 ± 0.2 g/kg/d. Calorie intake for male participants decreased from 1715 ± 284 kcal/day to 1571 ± 267 kcal/day, and for female participants from 1696 ± 231 kcal/day to 1451 ± 195 kcal/day. No adverse events were reported by the participants throughout the study.

### Acceptability of the intervention

Twenty-one participants (mean age = 68.19 ± 6.30 years old) from the experimental group attended the semi-structured interview. The adherence of the interviewees to keeping a food diary was diverse, from moderate to good (8/21) and bad (13/21). Four themes with corresponding sub-themes were synthesised from the data: (1) overall perceptions of the DBC intervention; (2) barriers to participating in the DBC intervention; (3) facilitators in implementing the DBC intervention; (4) suggestions for a future programme (see Table 5). The participants reported the DBC intervention was helpful for their health and they were motivated to change their dietary behaviour. The facilitators for their dietary behaviour change included the support from their family, the concerns about own health, the concerns about own body image, and the support from the researchers. However, some barriers for dietary behaviour change were also reported which included the barriers to keeping a food diary, the difficulties to estimate the food amount, yield to offspring’s taste, and overeat due to being unwilling to waste leftovers.

**Table 5:** Results of the individual semi-structured interview

Themes and sub-themes	Quotations
*1. Overall perceptions of the DBC intervention*	
1a. Helpful for one’s health.	“I feel (this course is) pretty good for my health. By attending the course, I have an idea of what calories are, and have been trying to control them a little bit. What you said inspires me.” (P2)
1b. Motivated to change dietary behaviour.	“Actually, I knew a little bit of nutrition knowledge before, but it is hard to change my dietary habits by myself. After taking your courses, I realise that I must change my unhealthy dietary habits.” (P11)
1c. Arrangements of the intervention sessions were acceptable.	“Recording the food makes my dietary patterns much clearer. For example, if I feel I have not eaten the meat for a long time, but I can’t recall how long, I just check the food diary.” (P1)
***2. Barriers to participating in the DBC intervention***	
2a. Barriers to keeping a food diary.	“Taking care of grandchildren has delayed a lot of things. I was too busy to sit down, so I missed recording the food very often.” (P6)
2b. Difficult to estimate the amount of food consumed.	“The packaged food, for example, a cake, I can put it on the scale and weigh it. But for a bowl of cooked rice, I can’t weigh it before eating and then weigh it after eating. It is a little difficult to practice.” (P5)
2c. Yield to offspring’s taste.	“We always consider the children’s appetite when we prepare the food, instead of ourselves.” (P20)
2d. Not willing to waste leftovers.	“We were poor when we were young, so I feel it is too wasteful to throw out the leftovers. I always eat them all. Now I am gradually starting to change my bad behaviour, like cooking less food to avoid leftovers.” (P3)
***3. Facilitators for implementing the DBC intervention***	
3a. Care about one’s health.	“Our children are not around. If my husband and I get sick, no one can take care of us. We have to take care of ourselves, so we care about the quality of our diets; it’s important for good health.” (P1)
3b. Support from family.	“My family supports me in controlling my diet. My husband was even worried that I would not insist on it. He continued to remind me.” (P21)
3c. Care about one’s body image.	“Because my body is fat without too much muscle, it is puffy. From the bottom of my heart, I want to get rid of the annoying fat.” (P5)
3d. Support from the diet instructor	“I have lost five or six catties in weight. I am lucky to meet you, and you gave me a lot of power.” (P9)
***4. Suggestions for a future programme: the content could be broader and deeper.***	“The cultural level of the older population in our generation is very different from ours, and it is good to deliver something that some people can easily understand. But for us who had higher education, the content could be more profound.” (P15)

### Preliminary effects of the intervention

Participants in the experimental group experienced a significant reduction in body weight (Wald χ2 = 4.90, p = 0.027, d = 1.22) and improvement in dietary quality (Wald χ2 = 12.66, p < 0.001, d = 1.31) after the intervention compared with those in the control group. A non-significant decrease in waist circumference (from 99.28 ± 1.32 cm to 98.42 ± 1.39 cm) and increase in handgrip strength (from 15.37 ± 1.08 kg to 18.21 ± 1.68 kg) and gait speed (from 0.91 ± 0.02 m/s to 0.99 ± 0.03 m/s) was observed within the experimental group from baseline to post-intervention. Reduction was observed in skeletal muscle mass index (from 7.31 ± 0.16 kg/m^2^ to 7.23 ± 0.19 kg/m^2^) in the DBC group, although this change shows no statistical significance. Table 6 showed the preliminary effects of the DBC intervention on outcomes over 15 weeks from baseline.

**Table 6:** Data analysis on study outcomes at baseline and post-intervention*

Outcomes	Mean (SE)		Baseline comparison		Group-by-time effect		Effect size
	Baseline	Post	*t/z*	*p*	Wald χ^2^	*p*	*d*
**Handgrip strength (kg)**			− .536^a^	0.592	1.96	0.162	0.45
DBC group	15.37 (1.08)	18.21 (1.68)					
Control group	14.47 (0.88)	15.56 (1.20)					
**Body weight (kg)**			1.38^b^	0.173	4.90	**0.027**	1.22
DBC group	75.28 (1.60)	74.78 (1.90)					
Control group	72.29 (1.40)	73.71 (1.62)					
**Waist circumference (cm)**			-1.09^b^	0.160	2.57	0.109	0.56
DBC group	99.28 (1.32)	98.42 (1.39)					
Control group	96.73 (1.17)	97.65 (1.53)					
**BMI (kg/m** ^**2**^ **)**			− .11^a^	0.912	1.54	0.214	0.40
DBC group	29.98 (0.62)	29.66 (0.67)					
Control group	29.77 (0.48)	29.93 (0.56)					
**Percentage of body fat (%)**			− .94^b^	0.351	0.75	0.388	0.25
DBC group	39.35 (1.09)	39.83 (1.25)					
Control group	40.70 (0.90)	40.11 (0.85)					
**Body fat mass (kg)**			− .44^a^	0.663	0.28	0.599	0.18
DBC group	29.22 (1.23)	29.77 (1.28)					
Control group	29.47 (0.97)	29.60 (1.00)					
**Skeletal muscle mass index (kg/m** ^**2**^ **)**			1.22^b^	0.280	3.45	0.063	0.84
DBC group	7.31 (0.16)	7.23 (0.19)					
Control group	7.05 (0.13)	7.16 (0.15)					
**SMM/weight (%)**			1.074^b^	0.287	2.71	0.100	0.56
DBC group	33.31 (0.65)	33.02 (0.79)					
Control group	32.38 (0.55)	33.04 (0.54)					
**6-m Gait speed (m/s)**			-1.69^a^	0.097	3.82	0.051	0.47
DBC group	0.91 (0.02)	0.99 (0.03)					
Control group	0.85 (0.03)	1.01 (0.03)					
**SPPB**			− .18^a^	0.859	1.18	0.278	0.31
DBC group	10.87 (0.22)	11.62 (0.13)					
Control group	10.67 (0.30)	11.08 (0.30)					
**MNA**			− .94^a^	0.347	0.08	0.780	0.33
DBC group	13.00 (0.30)	12.92 (0.19)					
Control group	13.43 (0.19)	13.25 (0.21)					
**Nutrition self-efficacy**			-1.33^a^	0.183	0.69	0.406	0.23
DBC group	15.20 (0.64)	16.69 (0.64)					
Control group	14.07 (0.68)	14.58 (0.80)					
**DQI-I (total)**			1.076^b^	0.286	12.66	**< 0.001**	1.31
DBC group	60.03 (2.02)	65.92 (2.35)					
Control group	56.83 (2.11)	57.83 (2.43)					
**SF-36**							
***Physical functioning***			− .52^a^	0.607	0.25	0.614	0.13
DBC group	81.00 (5.01)	88.08 (1.22)					
Control group	73.67 (3.15)	77.92 (2.45)					
***Role limitation due to physical problems***			− .25^a^	0.805	2.15	0.142	0.39
DBC group	80.83 (7.04)	95.19 (3.85)					
Control group	79.17 (7.08)	75.00 (8.46)					
***Bodily pain***			− .85^a^	0.393	0.74	0.389	0.23
DBC group	14.33 (3.01)	8.46 (1.86)					
Control group	17.00 (2.75)	14.58 (2.95)					
***General health***			− .39^a^	0.693	0.29	0.588	0.15
DBC group	54.50 (1.32)	50.00 (2.21)					
Control group	55.00 (1.33)	52.08 (1.52)					
***Vitality***			− .57^a^	0.572	0.25	0.618	0.14
DBC group	54.83 (2.29)	55.58 (1.20)					
Control group	52.17 (1.71)	54.58 (1.50)					
***Social functioning***			− .06^a^	0.955	2.05	0.152	0.39
DBC group	60.42 (1.57)	64.42 (1.62)					
Control group	60.83 (2.48)	59.90 (2.44)					
***Role limitation due to emotional problems***			− .56^a^	0.574	0.32	0.575	0.15
DBC group	82.22 (6.80)	92.31 (5.23)					
Control group	77.78 (7.26)	81.94 (7.60)					
***Mental health***			.38^b^	0.707	1.56	0.212	0.33
DBC group	55.73 (2.21)	60.00 (1.63)					
Control group	54.67 (1.69)	55.67 (1.27)					
***Reported health transition***			− .51^a^	0.613	1.36	0.243	0.32
DBC group	55.00 (2.98)	47.12 (3.92)					
Control group	57.50 (3.36)	56.25 (3.68)					

Notes: DBC = Dietary behaviour change; SE = standard error; BMI = body mass index; SPPB = short physical performance battery; MNA = mini nutritional assessment; DQI-I = Dietary Quality Index-International; SF-36 = the short form (36) health survey;

^a^ Mann-Whitney U test; ^b^ Independent Samples t-test

*The results were reported by adjusting the education level and body height

## Discussion

This study demonstrated that the DBC intervention is feasible and acceptable among the target population, as reflected by the relatively high attendance and retention rates, and by the positive feedback from the interviews. This study showed that the DBC could effectively reduce body weight and improve dietary quality among older adults with sarcopenic obesity. However, the effects on building muscles were nonsignificant.

### Feasibility and acceptability of the intervention

We screened around 2,000 people and only 105 people were eligible. The relatively low eligibility rate (5.25%) indicates that extensive screening may be needed in a future study. It is difficult to compare the eligibility rate in this study with those of previous interventional studies because the eligibility rate varied greatly among the different studies. For example, the eligibility rate in some studies could reach 62.36% [38], while in others it was only 3.21% or 7.62% [3, 39]. The variability could be due to a lack of standardisation in the diagnostic criteria for sarcopenic obesity, as various diagnostic criteria for screening the participants were used in previous studies. In addition, one previous investigation [40] showed that the prevalence of sarcopenic obesity among 101 males (aged 80 or above) in Beijing, China was 40.0% when using relative appendicular skeletal muscles for screening, and 95% when using a skeletal muscle mass index. In contrast, Du [41] reported that the prevalence of sarcopenic obesity was 7.0% in males and 2.4% in females after screening 213 males and 418 females in Shanghai, China, figures that were obtained using the old Asian criteria for sarcopenic obesity. A potential reason for the relatively low eligibility rate in this study compared to those of previous prevalence studies was that the older people were reluctant to visit the community health centres due to a fear of being infected by COVID-19. The collecting of data was suspended for six months due to the pandemic. We might not have obtained a comprehensive sample. In addition, the average age of the participants was 68.13 ± 6.12 years old. However, sarcopenia is more prevalent among the older population (i.e., 70 years or above) because muscle loss and fat accumulation are positively related to an increase in age [5]. This may have contributed to the higher prevalence found in previous cross-sectional studies (average age = 88.8 ± 3.7 years old) [40].

The findings of the semi-structured interview revealed both positive and negative aspects of the intervention process. On the one hand, the participants recognised the positive role of the BCTs in encouraging them to modify their dietary habits from unhealthy to healthy. The continuous support from the interventionist during the intervention process was reported as being a crucial factor in bringing about changes in behaviour. Previous studies on lifestyle modifications tended to focus on the provision of knowledge, materials, and professional education, which may be insufficient for making behavioural changes [42]. Instead, providing alternative strategies to deal with obstacles in actual practice, such as making the participants more aware of risky behaviour or facilitating their ability to self-monitor, might be more effective at changing behaviour [29].

Contextual cultural factors may pose some barriers to changing dietary behaviour, as reflected by the relatively low rate of adherence to keeping a food diary. According to our qualitative interviews, the barriers included internal factors (e.g., previous eating habits) and external factors (e.g., specific aspects of Chinese dining culture). For example, Chinese families are used to eating together and sharing dishes, which may cause difficulties for participants in controlling the amount of food that they eat compared to individual servings. In addition, some participants were busy taking care of their grandchildren, so they hardly had adequate time to keep a detailed food diary. These external barriers need to be addressed in future research by including family members in the study, to increase the awareness of family members and relieve some of the burden on the participants.

### Preliminary effects of the intervention

The findings on preliminary effects demonstrated that body weight could decreased significantly, with a simultaneous decrease in skeletal muscle. This preliminary finding is similar to that in previous interventional studies [16, 17], which showed a decrease in the lean body mass of older people associated with weight reduction after they had followed a hypocaloric diet for 3 or 4 months. Many studies have also reported the phenomenon of muscle loss along with weight reduction [5, 43, 44]. Obese adults could lose 2–10% of their muscle mass in a 8–10% diet-induced weight reduction [45–48]. There could be several reasons for this result. First, given the relatively small sample size in this pilot study, its statistical power might not have been sufficient to detect differences between the experimental and control groups [49]. Second, the duration of the intervention might have been insufficient to estimate the effects of dietary interventions on body composition. The preliminary results of this pilot study may indicate that a longer time is needed to see the effects in terms of muscle building.

On the other hand, we could observe a non-significant increase in handgrip strength and gait speed and the decrease in waist circumference in the intervention group from baseline to post-intervention. These findings are similar to those of previous nutritional studies [16, 17] in that a high-protein low-caloric diet led to a significant improvement in handgrip strength and decrease in waist circumference within the group after the intervention, even though the between-group effects when compared to those on a normal-protein low-caloric diet were not significant. The role of energy restriction and high protein intake have been proven to be extremely important for functional capacity improvement for healthy ageing [11]. Obesity and lack of activity in older people are strongly related to the physical dysfunction while the adjustment of energy and protein intake can help prevent the process [50].

### Limitations and strengths

There were some limitations to this study. First, it was challenging to perform double-blinding (blinding of the interventionist and participants) due to the nature of the study. Although we maintained continuous social contact with the control group to avoid the confounding effects of psychosocial contact, it was not possible to compensate entirely for the Hawthorne effect. Second, the method of assessing food intake in this study might have led to bias in estimating the amount of food that was consumed. Because the participants self-reported the amount of their food intake, there might have been variations between the actual amount and the estimated amount, even though the participants had been trained in measurement methods. However, the food diary is the most widely used method in current dietary intervention studies especially in a community-dwelling setting. We also considered using digital methods (e.g., technological equipment or application programs) to help in recording food intake. However, the accuracy of digital methods is yet to be established, and the problems of self-reporting remain unsolved [51]. Third, we did not conduct the process evaluation and explore the therapeutic mechanism, which could be addressed in a future full trial. Forth, the estimation of the sample size was referred to the rule of thumb without considering the attrition rate which did occur during the data collection. Finally, the interpretation of findings should be treated with caution because this is a pilot trial, but these pilot data can be used to power a future intervention.

This study has implications for both clinical practice and research. First, this study provides a good reference for community health providers to use to play a supervisory role in implementing dietary interventions using behaviour change techniques (e.g., workshops or telephone follow-ups), and then to improve the quality of the diets of older adults. Notably, the intervention in this study used an individualised rather than a uniform approach, which is crucial for dietary interventions considering the heterogeneity among participants in terms of lifestyle, mealtimes, confidence, and family context. Second, this study inspired the design for future research, i.e., a longer intervention duration and better-tailored methods for promoting compliance in the keeping of a food diary. In this pilot study, handgrip strength, waist-hip ratio, and gait speed all showed a non-significant positive change. Supposing these parameters could be significantly changed in a longer intervention duration and a bigger sample size.

## Conclusion

This pilot study supports the view that a dietary intervention combined with behaviour change techniques is a feasible and acceptable programme for older adults with sarcopenic obesity. The DBC intervention could reduce body weight, and has positive trends in managing handgrip strength, gait speed, and waist circumference. Interestingly, the subtle difference between the two groups in the change of muscle mass index warrants futures investigation. The effects of the DBC intervention on managing sarcopenic obesity could be further explored in a future study with a bigger sample size and longer intervention duration.

### Electronic supplementary material

Below is the link to the electronic supplementary material.

Supplementary Material 1

## Data Availability

The data and materials are not publicly availabe as the participants did not consenting to share their data. Further detials about the data and ethical conditions are available from the corresponding author on reasonable request.

## List of Abbreviations

DBC: Dietary behavioral change
HAPA: Health action process approach
RCT: Randomised controlled trial
RA: Research assistant
GEE: Generalized estimating equation
ITT: Intention-to-treat
BIA: Bioelectric impedance analysis
SD: Standard deviation
CI: Confidence interval
BMI: Body mass index
SPPB: Short physical performance battery
MNA: Mini-Nutritional Analysis
IPAQ-SF: International Physical Activity Questionnaire Short-form
DQI-I: Dietary quality index-international
